# Newborn screening for Fabry disease in the western region of Japan

**DOI:** 10.1016/j.ymgmr.2019.100562

**Published:** 2020-01-11

**Authors:** Takaaki Sawada, Jun Kido, Shinichiro Yoshida, Keishin Sugawara, Ken Momosaki, Takahito Inoue, Go Tajima, Hirotake Sawada, Shirou Mastumoto, Fumio Endo, Shinichi Hirose, Kimitoshi Nakamura

**Affiliations:** a Department of Pediatrics, Graduate School of Medical Sciences, Kumamoto University, Kumamoto, Japan; b KM Biologics Co., Ltd., Kumamoto, Japan; c Kumamoto-Ezuko Medical Center for Disabled Children, Kumamoto, Japan; d Departemnt of Pediatrics, School of Medicine, Fukuoka University, Fukuoka, Japan; e Departemnt of Pediatrics, Fukuoka University Nishijin Hospital, Fukuoka, Japan; f Division of Neonatal Screening, Research Institute, National Center for Child Health and Development, Tokyo, Japan; g Division of Pediatrics, Faculty of Medicine, University of Miyazaki, Miyazaki, Japan.

**Keywords:** α-Gal A, *GLA*, Fabry disease, Hypohidrosis, Newborn screening

## Abstract

Newborn screening (NBS) for Fabry disease (FD) is the best way to detect FD early prior to presentation of symptoms and is currently implemented in Taiwan and several states such as Illinois, Missouri, and Tennessee in the United States of America. In this report, we provide data from the first large-scale NBS program for FD in Japan. From August 2006 to December 2018, 599,711 newborns were screened; 26 variants, including 15 pathogenic variants and 11 variants of uncertain significance (VOUS; including eight novel variants), were detected in 57 newborns. Twenty-six male and 11 female newborns with pathogenic variants were diagnosed as hemizygous and heterozygous patients, respectively. Thirteen male and seven female newborns with VOUS were diagnosed as potential hemizygous and potential heterozygous patients, respectively. At the most recent follow up, three of 26 hemizygous patients had manifested symptoms and were receiving enzyme replacement therapy. The other patients were being followed up by clinicians. The frequency of FD (pathogenic variants + VOUS) in this study was estimated to be 1:7683, whereas that of patients with pathogenic variants was 1:11,854. In the future, the NBS system for FD may contribute to the detection of newborns not presenting manifestations related to FD and adults who have or have not developed manifestations related to FD.

## 1 Introduction

Fabry disease (FD; OMIM 301500) is an inherited X-linked glycosphingolipid storage disorder caused by mutations in the *GLA* gene, which encodes the lysosomal enzyme α-galactosidase A (α-Gal A, EC 3.2.1.22). Deficiency of α-Gal A results in progressive accumulation of metabolites, such as globotriaosylceramide (Gb3), in lysosomes and can cause progressive malfunctions in systemic organs, such as the skin, eyes, kidneys, ears, lungs, heart, and brain [[1], [2], [3]]. Male patients who have very low α-Gal A activity exhibit the classic phenotype and are generally asymptomatic until early childhood (the mean age of onset is reported to be 9 years) [4,5]. With advanced age, various clinical symptoms, including pain in the peripheral extremities, angiokeratoma, hypohidrosis, corneal opacity, renal disease, cardiac disease, and cerebrovascular disease, are developed, and premature death may occur.

Patients with FD having residual α-Gal A activity present milder clinical manifestations, and their onset time is later than that of patients with the classic type. Females with heterozygous mutations have a wide clinical manifestation spectra, ranging from asymptomatic to symptoms as severe as those in patients with the classic type, depending on random X-chromosomal inactivation [6,7]. To date, 516 variants have been incorporated into the public database (Fabry-database.org, last updated at February 15, 2019, ver.3.2.2) [8], and 349 of 516 variants were reported as a classic or severe type mutations.

Enzyme replacement therapy (ERT) is now approved for use in Japan, and three enzyme products (Fabrazyme, Replagal, and Agalsidase beta BS [JCR]) are available. ERT slows renal deterioration, alleviates the progression of cardiomyopathy, and prevents morbidity and death [9]. Several studies have demonstrated that ERT must be administered before the development of renal or cardiac failure in order to achieve optimal results [10,11]. Early treatment is critical to preserve organ function; however, many patients are diagnosed at later stages or misdiagnosed owing to the nonspecific clinical manifestations of the early stages of the disease [12].

Newborn screening (NBS) for FD is the best way to detect patients with FD at an early stage and who do not present manifestations; this approach is implemented in Taiwan and several states such as Illinois, Missouri, and Tennessee in the United States of America. Moreover, NBS can detect pediatric patients with FD who require early treatment immediately after onset. ERT can prevent further disease progression and improve significantly patient quality of life in pediatric patients with FD [13]. We performed NBS for 599,711 newborns in the western region of Japan from August 2006 to December 2018. In this report, we present the results of this NBS and discuss this NBS system and the genetic backgrounds of newborns with mutations or pathogenic variants.

## 2 Materials and methods

### 2.1 Study population

The study population consisted of 599,711 newborns born from August 2006 to December 2018, in six prefectures (Kumamoto, Fukuoka, Saga, Miyazaki, Kagawa, and Hiroshima) and two hospitals (University of the Ryukyus Hospital in Okinawa prefecture and Palmore Hospital in Hyougo prefecture; Supplementary Fig. S1) in Japan. The numbers of newborn were 212,228, 190,671, 16,799, 15,332, 38,447, 120,473, 2433, and 3328, respectively. Dried blood spots (DBSs) from newborns were prepared at maternity clinics or obstetric departments using standard procedures at 4–6 days after birth. After dropping blood spots onto filter papers (Toyo Roshi Kaisha, Ltd., Tokyo, Japan), the DBSs were dried for at least 4 h at room temperature, sent to Kumamoto University by mail, and if necessary, stored at −20 °C until use.

### 2.2 Flowchart of NBS for FD

**Fig. 1:**
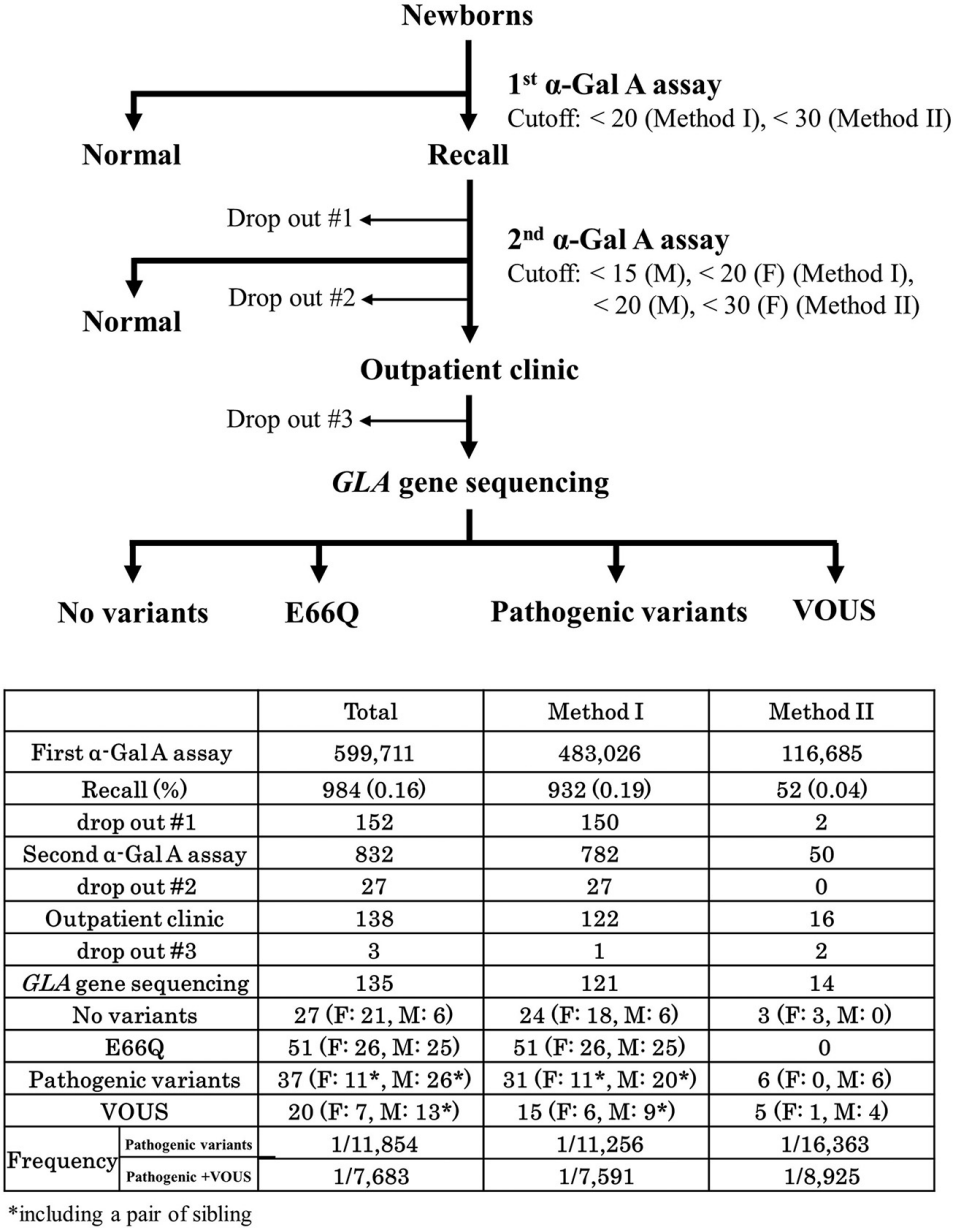
Flowchart of newborn screening for Fabry disease.

The NBS using α-Gal A activity assays with DBSs was performed to detect FD in three steps (Fig. 1). From August 2006 to October 2016, we performed α-Gal A assays with DBSs using Method I. From November 2016, we analyzed α-Gal A activity using Method II.

In the first step, newborns with α-Gal A activity under the cutoff level (< 20 [AgalU] for Method I, < 30 [AgalU] for Method II) were recalled, and their DBSs were prepared again. In the second step, newborns with α-Gal A activity under the cutoff level (Method I: < 15 for males, < 20 for females; Method II: < 20 for males, < 30 for females) were called to the hospital for clinical examinations. The newborns were examined using physical and biochemical assays to confirm symptomatic signs of FD, and *GLA* gene sequencing was performed in newborns whose parents provided informed consent.

### 2.3 α-Gal A assay

#### 2.3.1 Method I

The procedures for α-Gal A assays using DBSs were described in our previous report [14]. Briefly, a single 3.2-mm diameter disk punched from DBSs was incubated in a well of a 96-well clear microwell plate (Corning, NY, USA) with 40 μL Mcllvan buffer (100 mM citrate, 200 mM NaH_2_PO_4_, 36.8:63.2, pH 6.0) and processed for extraction at room temperature for 2 h. Aliquots of 30 μL blood extract were transferred to fresh 96-microwell plates. An aliquot of 100 μL of the reaction mixture (3.5 mM 4-methylumbelliferyl-α-d-galactopyranoside (4MU-αGal), 100 mM citrate, 200 mM K_2_HPO_4_, 100 mM *N*-acetyl-d-galactosamine) was added to each well of the microwell plates and incubated at 37 °C for 24 h. The reaction was terminated with 150 μL termination solution (300 mM glycine/NaOH, pH 10.6) immediately after the reaction. The fluorescence intensity from the 4-methylumbelliferones in the wells was measured with a fluorescence plate reader (BIO-TEK, Winooski, VT, USA) at 450 nm. One unit (1 AgalU) of enzymatic activity was equal to 0.34 pmol of 4MU-αGal cleaved/h per disc.

#### 2.3.2 Method II

Method II was developed for high-throughput and multiple assays by collaboration with KM Biologics Co., Ltd. (JP6360848B) and implemented from November 2016. Briefly, a single 3.2-mm diameter disk punched from DBSs was incubated in a well of a 96-well clear microwell plate (AS ONE Corporation, Osaka, Japan) with 100 μL of 25 mM citrate/potassium phosphate buffer (pH 6.0) containing 5 mM MgCl_2_, 0.5 mM dithiothreitol, 0.05% NaN_3_, and 0.1% Triton X-100 for 1 h at room temperature with gentle mixing. A 20-μL aliquot of the extract was then added to 40 μL of the reaction mixture (3.0 mM 4MU-αGal, 100 mM *N*-acetyl-d-galactosamine in 100 mM citrate/200 mM potassium phosphate buffer, pH 4.4) in a 96-well black microwell plate (Thermo Fisher Scientific Inc., MA, USA). The reaction mixture was incubated at 38 °C for 3 h, and the reaction was stopped by adding 200 μL of 300 mM glycine/NaOH buffer (pH 10.6) containing 10 mM ethylenediaminetetraacetic acid to measure fluorescence intensity. This residual extract could be used for the assay of acid α-glucosidase (Pompe disease) as well as glucocerebrosidase (Gaucher disease) activity.

### 2.4 Sequencing of the GLA gene

#### 2.4.1 Sanger method

Genomic DNA was extracted from total blood using a Gentra Puregene Blood Kit (Qiagen, Hilden, Germany) or equivalent product and stored at −80 °C until use. All seven exons and the flanking intronic sequences of the *GLA* gene were amplified by polymerase chain reaction (PCR) [15,16]. The sequence of intron 4 was also amplified to evaluate the mutation c.639 + 919G > A [17]. PCR products were sequenced on an ABI3500xl autosequencer (Applied Biosystems, Foster City, CA, USA) and analyzed using Sequencher 5.0 (Gene Codes Corporation, Ann Arbor, MI, USA).

#### 2.4.2 Next-generation sequencing (NGS)

**Supplementary Fig. S2:**
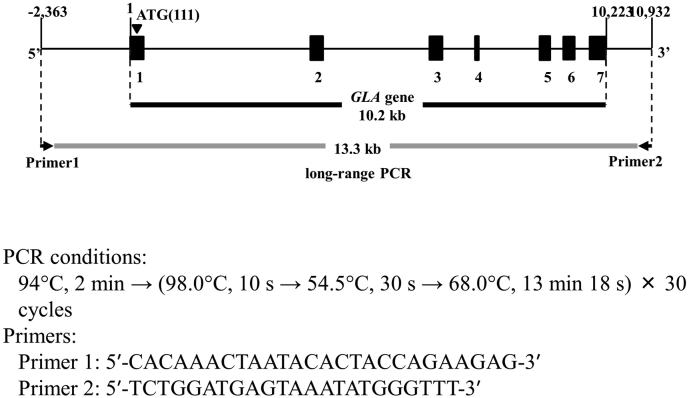
Long-range PCR of the *GLA gene*

Sequencing of the *GLA* gene by NGS was performed in collaboration with KM Biologics Co., Ltd. The procedures were described in our previous report [18]. Briefly, the 13.3-kbp region including the *GLA* gene was amplified by long-range PCR (Supplementary Fig. S2). Library preparation and sequencing were performed using a Nextera XT Kit (Illumina, San Diego, CA, USA) and MiSeq sequencer (Illumina). After sequencing runs, the data were aligned to the human reference genome sequence (NC_000023.10) using MiSeq Reporter software (Illumina). Sequence data analysis, mapping, and variant calling were streamlined using MiSeq Reporter v2 (Illumina). Visualization of sequencing reads was performed with IGV_2.3.10 (Broad Institute). Variants detected in the *GLA* gene by NGS were resequenced by the Sanger method.

### 2.5 Mutation analysis of the variants

The mRNA reference sequence RefSeq NM_000169.2 was used for this study; the “A” nucleotide of the ATG codon at nucleotide position 111 of the RefSeq sequence constituted +1 numbering of the cDNA sequence. The ATG codon also represented +1 for the amino acid numbering as set forth by the α-Gal A preprotein sequence NP_000160.1. Mutation nomenclature followed the guidelines established by the Human Genome Variation Society (http://varnomen.hgvs.org/). Public databases, including Fabry-database.org [8] (http://fabry-database.org/, updated February 15, 2019), and ClinVar [19] (http://www.ncbi.nlm.nih.gov/clinvar), were used for the classification of each variant. The software PolyPhen-2 [20] (http://genetics.bwh.harvard.edu/pph2) was used for missense mutations to predict the potential impact of an amino acid alteration on the function of α-Gal A.

### 2.6 Statistical analyses

The α-Gal A activities obtained from Methods I and II were compared between male and female newborns using *t*-tests. Statistical analyses were performed using IBM SPSS software, version 25 (IBM Corporation, Armonk, NY, USA). Results with *P* values of <0.05 were considered statistically significant.

### 2.7 Ethics

This study was approved by the Ethics Committee of Kumamoto University (approval no. 1537). Written informed consent was obtained from the parents or legal guardians of newborns.

## 3 Results

### 3.1 NBS for FD

**Fig. 2:**
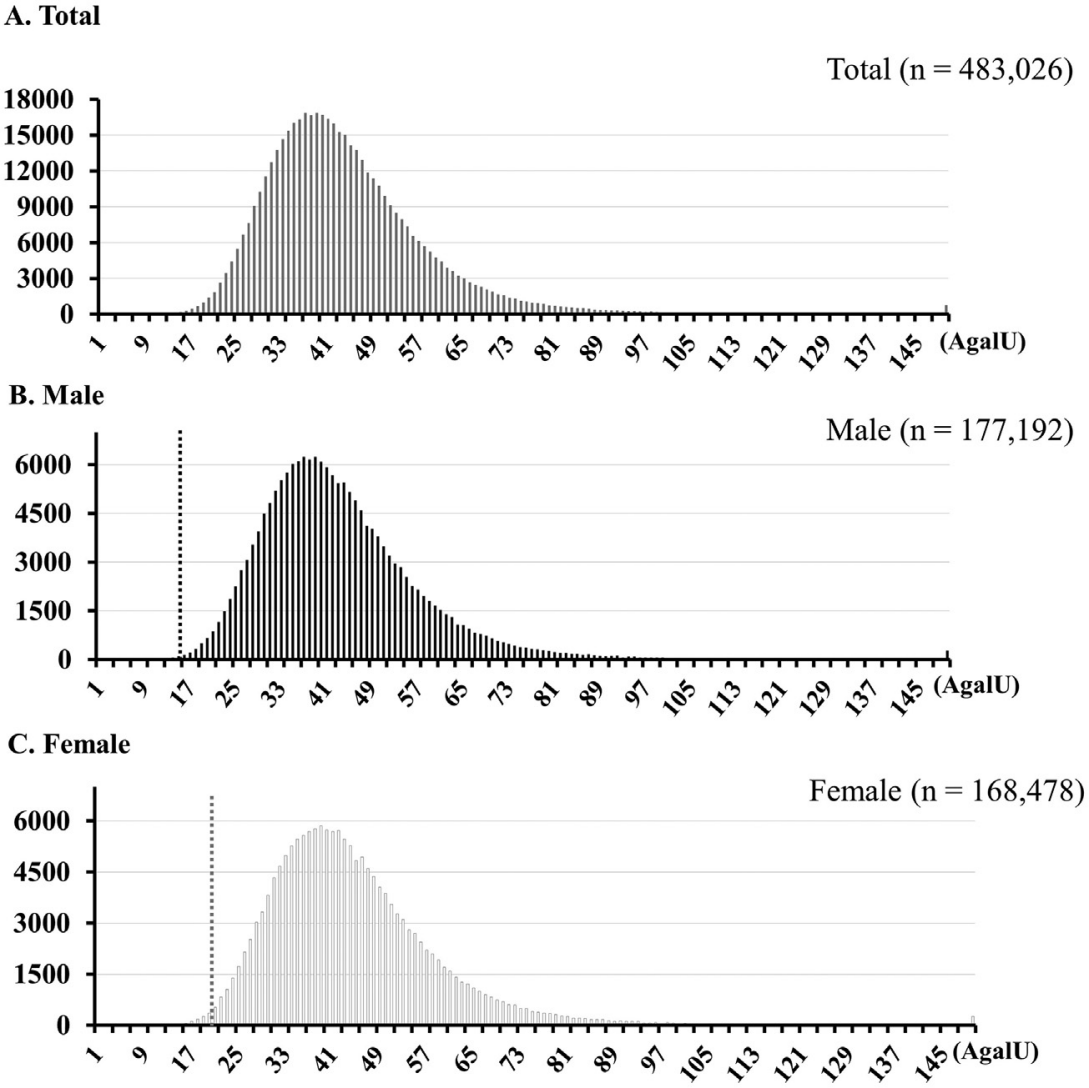
Histograms of α-Gal A activity in newborns. Histograms of α-Gal A activity are shown for (A) the total population of newborns (*N* = 483,026), (B) male newborns (*N* = 177,192), and (C) female newborns (*N* = 168,478). Dash line: cutoff level.

The flowchart and results for the NBS program for FD are shown in Fig. 1. In total, 599,711 newborns (483,026 in Method I and 116, 685 in Method II) were screened. The mean and median α-Gal A activities obtained using Method I were 43.54 ± 14.93 (AgalU) and 41.50 (AgalU; interquartile range [IQR]: 34.22–50.51), respectively, in male newborns and 45.03 ± 15.01 (AgalU) and 42.63 (AgalU; IQR: 35.26–51.72), respectively, in female newborns (Fig. 2). The mean and median activities obtained using Method II were 128.09 ± 59.82 (AgalU) and 118.18 (AgalU; IQR: 85.82–158.88), respectively, in male newborns and 134.15 ± 61.56 (AgalU) and 124.09 (AgalU; IQR: 90.88–165.74), respectively, in female newborns. The α-Gal A activities of female newborns were slightly higher than those of male newborns in both Method I and Method II (*p* < .001 and *p* < .001, respectively).

Next, 984 (0.16%) newborns were recalled for the second α-Gal A assay, and 138 newborns with low α-Gal A activities under the cutoff at the second α-Gal A assay were examined at outpatient clinics. These newborns were evaluated using physical and laboratory examinations, and *GLA* gene sequencing was performed for 135 newborns. Twenty-seven newborns presented with no variants in the *GLA* gene, whereas 51 newborns presented with a functional polymorphism allele, p.E66Q [21]. Fifty-seven newborns, including three siblings, presented with pathogenic variants or variants of unknown significance (VOUS). The frequency of FD (pathogenic variants + VOUS) in this study was estimated to be 1:7683 (0.013%), whereas that of pathogenic variants was 1:11,854 (0.008%). The frequency of male FD was estimated to be 1:6212 (0.016%). These data were comparable to the results of our previous pilot study, in which we detected pathogenic variants with a frequency of 1:10,585 [14].

### 3.2 Variants detected in newborns

The *GLA* gene is highly polymorphic, and many novel mutations are still being discovered. At present, 426 and 516 variants are registered in ClinVar and Fabry-database.org, respectively. In this study, 26 variants were detected in 57 newborns (Table 1). Twenty-four of 26 variants were missense mutations, and the remaining two were in-frame deletions and intronic mutations. Fifteen of these 26 variants were registered in ClinVar or Fabry-database.org. Three variants, i.e., c.725 T > C (p.I242T), c.1072_1074delGAG (p.E358del), and c.1171A > G (p.K391E), were reported by Sakuraba et al. in a Japanese patient [22], by Monserrat et al. in a 50-year-old male patient [23], and by Wakakuri et al. in a 29-year-old female patient [24], respectively. The other eight variants, i.e., c.428C > T (p.A143V), c.493G > A (p.D165N), c.538 T > G (p.L180 V), c.605G > T (p.C202F), c.625 T > C (p.W209R), c.685 T > G (p.F229 V), c.714 T > A (p.S238R), and c.1231G > A (p.G411S), were novel. We classified 15 of 26 variants as pathogenic variants and the other 11 variants, including eight novel variants, as VOUS.

**Table 1:** Variants detected in newborn screening for Fabry disease.

Variant No.	Nucleotide change	Amino acid change	Location	Databases		PolyPhen-2 (Score)	Amenability**	Classification
				ClinVar	Fabry-database.org*			
1	c.2 T > A	p.M1K	Exon 1	NR	Dobrovolny (2008)	Benign (0.080)	−	Pathogenic (classic)
2	c.124A > G	p.M42 V	Exon 1	Pathogenic	Park (2009)	Probably damaging	+	
3	c.128G > A	p.G43D	Exon 1	NR	Sakuraba (1990)	Probably damaging	−	
7	c.431G > A	p.G144D	Exon 3	NR	Li (2014)	Probably damaging	+	
11	c.595G > A	p.V199 M	Exon 4	Uncertain significance	Shabbeer (2002)	Probably damaging	+	
23	c.1072_1074delGAG	p.E358del	Exon 7	NR	NR	−	−	
25	c.1208 T > C	p.L403S	Exon 7	NR	Shimotori (2008)	Probably damaging	+	
4	c.290C > T	p.A97V	Exon 2	pathogenic	Eng (1997)	Possibly damaging (0.882)	+	Pathogenic (non-classic)
5	c.335G > A	p.R112H	Exon 2	pathogenic	Shimotori (2008)	Probably damaging	+	
8	c.436C > T	p.P146S	Exon 3	pathogenic	Mild, late-onset	Probably damaging	+	
14	c.628C > T	p.P210S	Exon 4	NR	Eng (1994)	Possibly damaging (0.758)	+	
15	c.639 + 919G > A	−	Intron 4	pathogenic	NR	−	?	
16	c.644A > G	p.N215S	Exon 5	pathogenic	Ebrahim (2012)	Benign (0.048)	+	
18	c.687 T > G	p.F229 L	Exon 5	NR	Kawano (2007)	Possibly damaging (0.950)	+	
21	c.888G > A	p.M296I	Exon 6	pathogenic	Saito (2013)	Benign (0.309)	+	
6	c.428C > T	p.A143V	Exon 3	NR	NR	Probably damaging	NA	VOUS
9	c.493G > A	p.D165N	Exon 3	NR	NR	Probably damaging	NA	
12	c.605G > T	p.C202F	Exon 4	NR	NR	Probably damaging	NA	
17	c.685 T > G	p.F229 V	Exon 5	NR	NR	Probably damaging	NA	
19	c.714 T > A	p.S238R	Exon 5	NR	NR	Probably damaging	−	
26	c.1231G > A	p.G411S	Exon 7	NR	NR	Probably damaging	NA	
10	c.538 T > G	p.L180 V	Exon 3	NR	NR	Possibly damaging (0.470)	NA	
24	c.1171A > G	p.K391E	Exon 7	NR	NR	Benign (0.266)	+	
22	c.1067G > A	p.R356Q	Exon 7	Conflicting interpretations of pathogenicity	Hwu (2009)	Benign (0.190)	+	
20	c.725 T > C	p.I242T	Exon 5	NR	NR	Benign (0.079)	+	
13	c.625 T > C	p.W209R	Exon 4	NR	NR	Benign (0.000)	NA	

*last updated February 15, 2019 (ver.3.2.2), **Summary of product characteristics of Galafold®, Table 2, Table 3. +: amenable, −: not amenable,?: unknown, NA: not available.

NR: not registered, VOUS: variants of uncertain significance: Novel variatns.

Table 2 shows the variants detected in the 57 newborns (39 males and 18 females). The most common variants in the 57 newborns were c.725 T > C/p.I242T (allele frequency: 8.45%) and c.888G > A/p.M296I (8.45%). The second most common variants were c.335G > A/p.R112H (7.04%), c.436C > T/p.P146S (7.04%), and c.639 + 919G > A (7.04%), followed by c.625 T > G/p.W209R (4.23%). Twenty-six male newborns and 11 female newborns having pathogenic variants were diagnosed as hemizygous and heterozygous patients, respectively. Additionally, 13 male newborns and seven female newborns having VOUS were diagnosed as potential hemizygous and potential heterozygous patients, respectively.

**Table 2:**
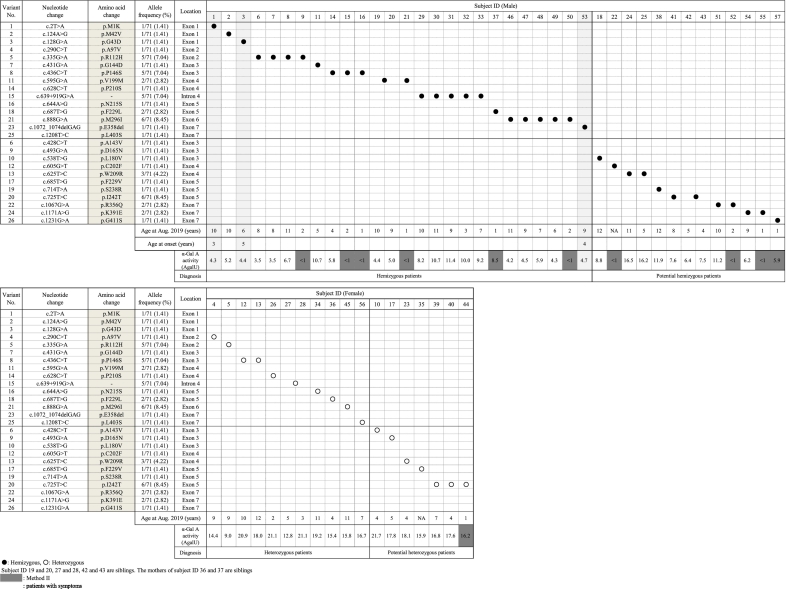
Patients with pathogenic or potential pathogenic variants detected in newborn screening for Fabry disease.

### 3.3 Follow-up survey for the 57 newborns

Fifty-seven newborns received follow-up and intervention by clinicians. Three hemizygous patients already had presented symptoms and received ERT (Table 3). Patient no. 1 developed extremity pains when taking a bath and hypohidrosis at the age of 3 years and was treated by ERT beginning at 5 years of age. For patient no. 3, follow-up cardiac magnetic resonance imaging showed delayed myocardial enhancement findings at the age of 5 years [25], and Mulberry cells were also detected in urinary sediment. Patient no. 53 had developed extremity pain and hypohidrosis at 4 years of age. Although the pain was reduced by ERT, it was exacerbated at 8 years of age, and the patient underwent carbamazepine treatment.

**Table 3:** Summary of patients who were symptomatic for Fabry disease.

Patient ID	Gender	Mutation		Age at Aug. 2019	Age of onset	Symptoms at onset	Treatment
		nucleic acid	amino acid				
1	M	c.2 T > A	p.M1K	10y2m	3y	hypohidrosis, extremity pain at bath	ERT from 5y
3	M	c.128G > A	p.G43D	6y7m	5y	delayed enhanced cardiac MRI, Mulberry cells in urinary sediment	ERT from 6y
53	M	c.1072_1074 delGAG	p.E358del	9y10m	4y	extremity pain, hypohidrosis	ERT from 6y, carbamazepine from 8y

## 4 Discussion

In this NBS program for FD, 57 newborns from 54 families who had pathogenic variants or VOUS were detected. The frequency of FD in this study was estimated to be 1:7683 (0.013%), whereas that of pathogenic variants was 1:11,854 (0.008%). The frequency of male FD was estimated to be 1:6212 (0.016%).

These findings were comparable to the results of our previous pilot study [14] and other NBS studies performed in WA [26], IL [27], and NY in the USA [28]; the prevalence rates of FD with pathogenic variants were 1:10,585 (2/21,170), 1:18,151 (6/108,905), 1:21,973 (10/219,730), and 1:9373 (7/65,605) live births, respectively. The frequencies of male FD were 1:1200–1:1400 in Taiwan [29,30], 1:4600 in Italy [31], and 1:7800 in WA, USA [26], respectively. Thus, the prevalence of FD in the Taiwan Chinese population is relatively high. In Taiwan, the non-classic type variant c.639 + 919G > A has an extremely high incidence owing to the high prevalence of 1:2810 (282/792,247) with pathogenic variants, as reported previously [32]. The allele frequencies of c.639 + 919G > A in newborns with variants from Taiwan and Japan (in this study) were considerably different (81.6% and 7.04%, respectively) in this study.

The mutation spectra of *GLA* in Japanese patients were reported previously [22,33]. *GLA* gene analysis was performed for 207 patients with FD, and 83 pathogenic variants were detected [22]. The most common variant was c.888G > A/p.M296I (12/207), followed by c.639 + 919G > A (9/207), c.679C > T/p.R227* (9/207), c.334C > T/p.R112C (8/207), c.335G > A/p.R112H (8/207), and c.902G > A/p.R301Q (8/207). In another study, 73 pathogenic variants were detected from 176 patients in 115 families [33]; the most common variant was c.334C > T/p.R112C (allele frequency: 2.65%), followed by c.888G > A/p.M296I (1.89%), c.658C > T/p.R220* (1.52%), c.718_719delAA (1.52%), and c.1025G > A/p.R342Q (1.52%). Even in Japan, there are differences in the genetic backgrounds of the *GLA* gene between the Kanto and Kyushu regions. Moreover, Kobayashi et al. demonstrated the prevalence of classical type (classical:late-onset type = 7:1, *N* = 88) in male patients with FD [33]. They reported the high frequency of classical type variants in male Japanese patients. However, this NBS study demonstrated that 28% of male newborns with pathogenic variants (7/25 families) and 19% of male newborns with pathogenic or VOUS variants (7/37 families) presented classical type variants. In the study by Kobayashi et al., they investigated variants in patients from Japanese families with FD at their institution and considered two patients with the same classical type variant in the same family as two variants. Because there were significant biases for patients analyzed in their study, the results in this study differed from those of their study.

Identification of pathogenic and late-onset variants by NBS and follow-up are essential for proper therapeutic intervention. At the follow up, three hemizygous patients had developed symptoms. These cases were detected by NBS at a presymptomatic stage and received proper therapeutic intervention. The other 54 patients are still undergoing follow up and intervention.

Some members in the proband's family were diagnosed with FD from the proband's diagnosis and started receiving ERT. For all three patients receiving ERT, their mothers were found to harbor the same pathogenic variants as their children. Because the mothers had already developed manifestations related to FD, they also underwent ERT. Moreover, the maternal grandfather of patient no. 11 was also diagnosed with FD and received ERT. Therefore, NBS contributed to detecting FD newborns without clinical manifestations of FD as well as new adult patients with FD who had not yet been diagnosed and had not undergone treatment for FD.

In this study, two types of α-Gal A assay methods, i.e., Method I and Method II, were utilized. Method II was developed for multiple and high-throughput assays and could be used to screen for Pompe disease, Gaucher disease, and FD simultaneously using a single 3.2-mm diameter disk. In Method II, because the extraction time and reaction time could be dramatically shortened, high-throughput assays for measurement of α-Gal A activity in DBSs could be performed within 8 h.

Method II showed a lower detection frequency for pathogenic variants and did not detect p.E66Q in any patient. The cutoff levels set for Method II should be reconsidered because the recall rate in Method II was lower than that in Method I, and the current Method II system using this cutoff may miss patients with pathogenic variants or p.E66Q.

This study had a number of limitations. First, in Method II, some patients with levels greater than the cutoff may harbor pathogenic variants or p.E66Q. The cutoff levels in the NBS system have already been evaluated several times; however, it is difficult to decide the best cutoff level when considering sensitivity, specificity, and cost performance. Second, the time at which NBS for FD was started differed from each area included in this study. A pilot study of NBS for FD was started in Kumamoto in 2006, and newborns participating in this NBS were gradually increased, while the area in which NBS was performed gradually expanded. Currently, NBS for FD is performed only in a limited region of Japan. In the future, we hope that NBS for FD and other lysosomal storage diseases, such as Gaucher disease and Pompe disease, will be performed throughout Japan as a public service.

In conclusion, we performed NBS for FD in 599,711 newborns and detected 15 pathogenic variants and 11 variants, including eight novel VOUS. The *GLA* gene mutation locus, which we detected in the western region of Japan and had been reported in patients with FD from the eastern regions of Japan and Taiwan, was different. Moreover, the prevalence of FD in Japan was significantly lower than that in Taiwan. These results may reflect differences in genetic backgrounds between the western and eastern regions of Japan and between Japanese and Taiwanese populations. In the future, our NBS system for FD could contribute to detection of newborns not presenting manifestations of FD and adults who have or have not developed manifestations related to FD.

**The following are the supplementary data related to this article.Supplementary Fig. S1:**
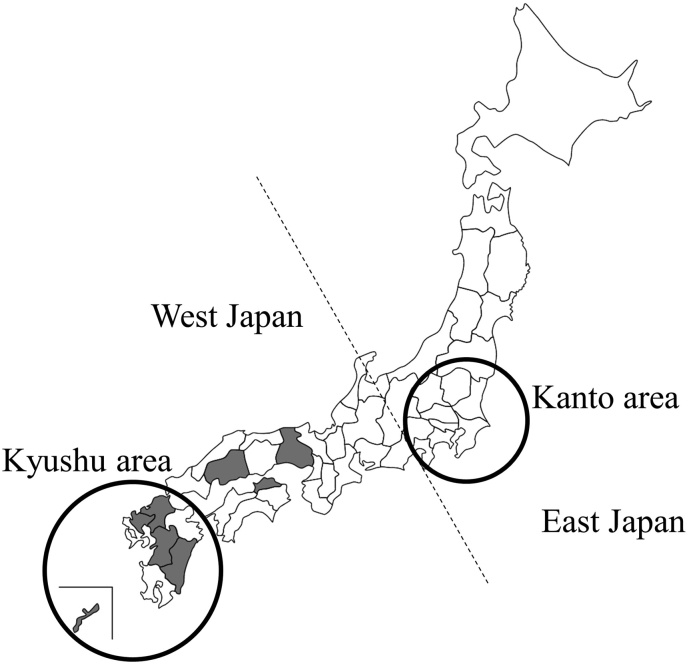
Areas participating in the NBS for FD in Japan

## Declaration of Competing Interest

All authors declare that there are no conflicts of interest associated with this study.
